# The erector spinae plane block for effective analgesia after lung lobectomy: Three cases report

**DOI:** 10.1097/MD.0000000000016262

**Published:** 2019-07-19

**Authors:** Seunguk Bang, Kyudon Chung, Jihyun Chung, Subin Yoo, Sujin Baek, Sang Mook Lee

**Affiliations:** a Department of Anesthesiology and Pain Medicine, Daejeon St. Mary's Hospital, College of Medicine, The Catholic University of Korea, Daejeon; b Department of Anesthesiology and Pain Medicine, College of Medicine, The Catholic University of Korea, Seoul, Republic of Korea.

**Keywords:** analgesia, catheter, erector spinae plane block, nerve block, pain, pneumonectomy, ultrasonography

## Abstract

**Rationale:**

The thoracic epidural block and thoracic paravertebral block are widely used techniques for multimodal analgesia after thoracic surgery. However, they have several adverse effects, and are not technically easy. Recently, the erector spinae plane block (ESPB), an injected local anesthetic deep to the erector spinae muscle, is a relatively simple and safe technique.

**Patient concerns:**

Three patients were scheduled for video assisted thoracoscopic lobectomy with mediastinal lymph node dissection. All the patients denied any past medical history to be noted.

**Diagnoses:**

They were diagnosed with primary adenocarcinoma requiring lobectomy of lung.

**Interventions:**

The continuous ESPB was performed at the level of the T5 transverse process. The patient was received the multimodal analgesia consisted of oral celecoxib 200 mg twice daily, intravenous patient-controlled analgesia (Fentanyl 700 mcg, ketorolac 180 mg, total volume 100 ml), and local anesthetic (0.375% ropivacaine 30 ml with epinephrine 1:200000) injection via indwelling catheter every 12 hours for 5 days. Additionally, we injected a mixture of ropivacaine and contrast through the indwelling catheter for verifying effect of ESPB and performed Computed tomography 30 minutes later.

**Outcomes:**

The pain score was maintained below 3 points for postoperative 5 days, and no additional rescue analgesics were administered during this period. In the computed tomography, the contrast spread laterally from T2-T12 deep to the erector spinae muscle. On coronal view, the contrast spread to the costotransverse ligament connecting the rib and the transverse process. In the 3D reconstruction, the contrast spread from T6-T10 to the costotransverse foramen.

**Lessons:**

Our contrast imaging data provides valuable information about mechanism of ESPB from a living patient, and our report shows that ESPB can be a good option as a multimodal analgesia after lung lobectomy.

## 1 Introduction

The thoracic epidural block (TEB) and thoracic paravertebral block (TPVB) are the most commonly used techniques for analgesia after thoracic surgery.^[1–3]^ However, TEB has several adverse effects, such as hypotension, motor blockade, hematoma, and abscess and TPVB has a chance of epidural spread and pneumothorax, and multiple injections are needed if more than 4 dermatome analgesia is required.^[1,2,4–6]^ Neither TEB nor TPVB are technically easy.^[7]^

Recently, erector spinae plane block (ESPB) was reported as a treatment for thoracic neuropathic pain.^[8]^ ESPB is a relatively simple technique with easily identified sonographic landmarks, and a catheter is easily inserted into the plane after distention induced by the injection.^[9,10]^ Additionally, the ESPB has the potential to provide both somatic and visceral sensory blockade.^[11]^ We report three cases of video-assisted thoracoscopic (VATS) lobectomy in which continuous ESPB were used to provide highly effective analgesia, and the effect of ESPB was verified by radiologic evaluation in a living patient.

## 2 Case report

Informed written consent for the continuous ESPB procedure and publication of this report were obtained from the patients. This report was approved by the Catholic University Hospital Institutional Review Board, Daejeon, Korea (DC18ZESI0001).

### 2.1 Case 1

**Figure 1:**
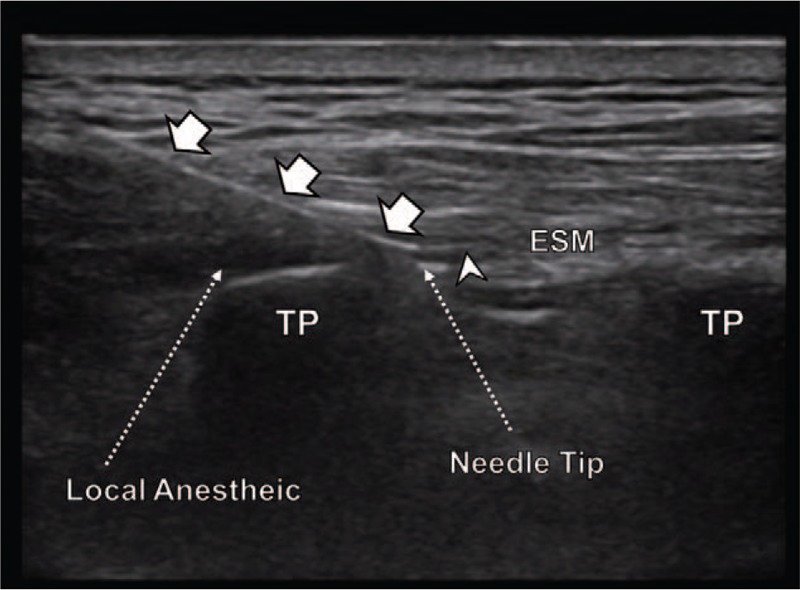
Ultrasound guided erector spinae plane block. Tuohy needle was inserted in a cephalad-to-caudal direction, toward the 3 muscles and the transverse process of T5. After contacting the transverse process, the needle was advanced forward. After confirming that the needle was deep to the erector spinae muscle, we injected the local anesthetic. Then, a 19-gauge epidural catheter was inserted using real time ultrasound guidance. Arrow head = catheter, arrow = needle, ESM = erector spinae muscle, TP = transverse process.

A 57-year-old woman (46 kg, 146 cm) was scheduled for VATS lobectomy due to primary adenocarcinoma. The continuous ESPB was performed at the level of T5 transverse process. The level of the rib and transverse process was located using ultrasound and a counting-down approach from the first rib. After placing a 5 to 12 MHz linear probe parallel to the vertebral axis, we found the T5 transverse process and 3 associated muscles (trapezius, rhomboid major, erector spinae muscle). From this point, an 18G Tuohy needle attached to an intravenous extension tubing between the needle and the syringe was inserted toward the 3 muscles and the transverse process of T5 in a cephalad-to-caudal direction. After confirming that the needle was deep to the erector spinae muscle using 2 ml of saline, we injected the prepared mixture of 0.75% ropivacaine (15 ml) with saline (15 ml). Then, a 19-gauge epidural catheter was inserted over 2 cm using real time ultrasound guidance, and placement was confirmed by saline injection (Fig. 1). After tagging suture, right lower lobectomy was performed using three thoracoscopic port incisions and one working window.

The patient received intravenous ketorolac 30 mg and fentanyl 50 mcg at the end of surgery. Postoperative multimodal analgesia consisted of oral celecoxib 200 mg two times per day, combined with intravenous patient-controlled analgesia (PCA) and local anesthetic injection via catheter according to the acute pain service protocol of our hospital. The PCA was programmed to deliver a bolus dose of 3 ml and background infusion of 1 ml, (fentanyl 1000 mcg, ketorolac 180 mg, total volume 100 ml). A bolus dose of 0.375% ropivacaine 30 ml with epinephrine (1:200000) was injected via indwelling catheter by manual injection every 12 hours for 5 days. Resting and dynamic (coughing, deep breathing) pain scores after surgery were assessed by using the visual analogue scale (VAS) score. If the resting VAS was more than 4 points, intravenous tramadol (25 mg) was prescribed as rescue analgesia.

Immediately after transfer to the intensive care unit, the patient's vital signs were stable and resting VAS was evaluated (resting 3, dynamic noncheckable). Two hours after surgery, the VAS scores of resting/deep breathing/active coughing were 1/1/3. At that time, the dermatome of sensory blockade using pinprick was T2-8 on the mid axillary line. Twelve hours after surgery, the patient complained of nausea; PCA was stopped and removed. The patient's postoperative pain remained well controlled without PCA. The resting/dynamic pain scores were kept below 3 points from 12 hours to 36 hours after surgery, and resting/dynamic pain score was maintained at 0-1 after 36 hours postoperative. No additional rescue analgesics were administered during this period.

**Figure 2:**
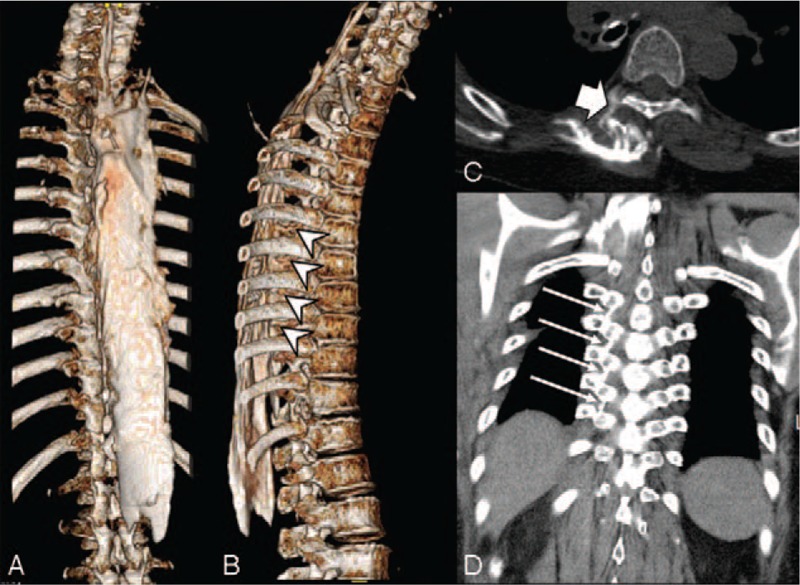
Computed tomography scan and 3-dimensional reconstruction. (A) The contrast spread extensively in the cephalocaudal direction between the C4 and L1 vertebrae. (B) Arrow head indicate that the contrast spread to the costotransverse foramen at level of T6-T10. (C) Contrast spread to the thoracic paravertebral space (thick arrow). (D) On the coronal section, the contrast spread to the costotransverse ligament (arrows), which connects the rib and the transverse process.

At postoperative day 6, we explained the excellent analgesic properties of ESPB to the patient again, and acquired permission and informed consent for computed tomography (CT) scan and publication of the report. We injected a mixture of 0.75% ropivacaine (10 ml), saline (10 ml), and contrast (10 ml) (VISIPAQUE, GE Healthcare, Cork, Ireland) through the indwelling catheter, and performed 3D CT 30 minutes later. Images demonstrated extensive cephalocaudal injection spread between the C4 and L1 vertebrae with medial spread toward the midline but limited lateral spread (Fig. 2). The contrast did not cross over the spinous process of the spine on the medial side; the contrast spread laterally from T2 -T12 deep to the erector spinae muscle. On coronal view, the contrast spread to the costotransverse ligament connecting the rib and the transverse process. In the 3D reconstruction, the contrast spread from T6-T10 to the costotransverse foramen.

The patient was very satisfied with postoperative pain control, and there was no remnant pain at the surgical site 2 weeks after surgery. She was discharged without any complications.

### 2.2 Case 2

A 72-year-old woman (52 kg, 152 cm) underwent VATS lobectomy of right upper lung. Unlike case 1, ultrasound-guided ESPB was performed at the end of the surgery. After ESPB procedure, fentanyl 50 μg and ketorolac 30 mg were injected, and patient was allowed to recover from general anesthesia. Postoperative multimodal analgesia consisted of oral celecoxib 200 mg twice daily, intravenous PCA (Fentanyl 700 mcg, ketorolac 180 mg, total volume 100 ml; background infusion 1 ml/h, bolus 3 ml/h), and local anesthetic injection via indwelling catheter (bolus 0.375% ropivacaine 30 ml with epinephrine 1:200000) every 12 hours for 5 days, as in Case 1. The resting/dynamic VAS score was kept below 3 points for 5 days after the surgery; no additional postoperative rescue drugs were required in the period. The follow-up at 2 weeks post operation were uneventful.

### 2.3 Case 3

A 54-year-old woman (49 kg, 155 cm) underwent VATS lobectomy of the right middle lobe with mediastinal lymph node dissection. At the end of surgery, ultrasound-guided ESPB was performed; postoperative multimodal analgesia was performed according to the acute pain service protocol of our hospital, as in Cases 2. The pain resting/dynamic VAS score was maintained between 1 and 2 for a week after surgery. No rescue drugs were administered during the postoperative period. After 2 weeks of follow-up, no neurologic symptoms were reported.

## 3 Discussion

The ESPB is a fascial plane block in which local anesthetic is injected deep to the erector spinae muscle. The target points - the transverse process and erector spinae muscle - are easily visualized on ultrasound.^[8–10]^ They are distant from the pleura and neuraxis, thus decreasing the risk of complications associated with injury to these structures. Local anesthetic spreads readily in this tissue plane and the volume of 20 to 30 mL in adults produces extensive cephalocaudal spread and anesthesia of several dermatomes.^[12–16]^ Studies advocate the volume-dependent spread of the local anesthetics in the ESPB. Damjanovska et al^[17]^ performed a retrolaminar injection to discover that the spread of the injectate to the ipsilateral paravertebral space was only observed in his high-volume group (30 ml) as opposed to his low-volume group (10 ml). As the retrolaminar block, the clinical effects of the ESPB rely on the spread of the local anesthetic. Therefore, it is legitimate to deduce that the injectate volume of the ESPB should also be considerable. Cadaveric studies reported to perform the ESPB at the T5 level with 20 ml of dye to achieve the spread in the paravertebral and intercostal spaces from T2 to T10.^[18]^ Also, Freo et al^[8]^ reported that ultrasound-guided ESPB provided an acquired sensory blockade from T3 to T9 over the posterior thorax and T3 to T6 over the anterolateral thorax after injection of 0.5% ropivacaine (20 ml) at the T5 level, and reported that ESPB was a good option for analgesia after video-assisted thoracoscopic lobectomy. In another report, after injection of 0.5% ropivacaine (30 ml) at the T9 level, loss of pinprick from T2 to T10 was acquired.^[19]^ Another study, however, reported to partially influence up to C7 and C8 dermatomes with 35 ml of the local anesthetic.^[20]^

**Figure 3:**
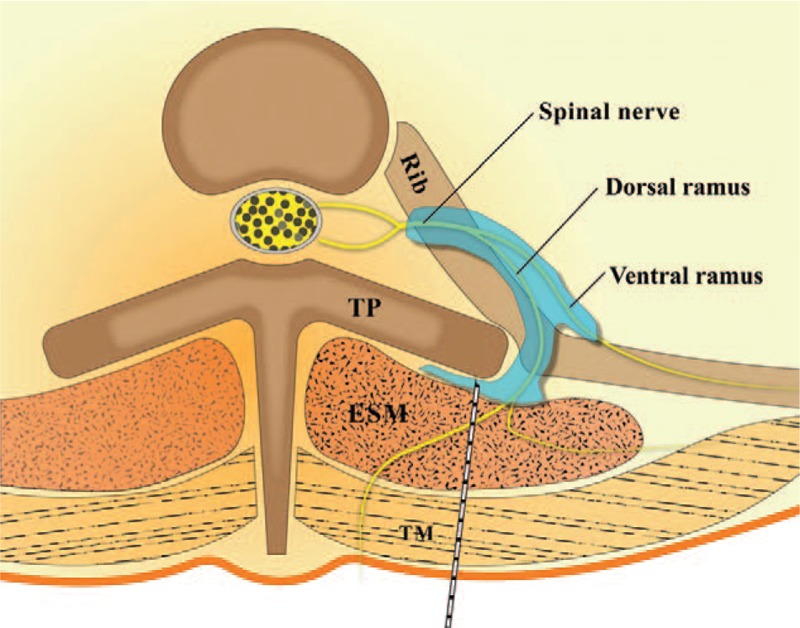
Schematic diagram. Local anesthetic, which is injected deep to the erector spinae muscle, spreads to the thoracic paravertebral and intercostal space through the fenestrations in the costotransverse ligament and costotransverse foramen. Image adapted and used with permission from Journal of Korean Medical Science.^[9]^ ESM = erector spinae muscle, TM = trapezius muscle, TP = transverse process.

We assume that this technique blocks both dorsal ramus and ventral ramus of the spinal nerve through the intercostal space and thoracic paravertebral space (Fig. 3). We think the mechanism is 2-fold: spread through the bony gap, and penetration through porous tissue around the superior costotransverse ligament. A cadaveric study showed that the dorsal and ventral ramus of spinal nerve was stained by contrast dye injected deep into the erector spinae muscle.^[8]^ The dye spread to the thoracic paravertebral and intercostal spaces through a bony gap, such as the costotransverse foramen.^[8,18]^

Conventionally, the superior costotransverse ligament was assumed to be a compact ligament and effective TPVB could only be achieved after penetration of the ligament. However, Costache et al^[21]^ showed that even with the injection of the dye above the superior costotransverse ligament or intercostal muscle, the thoracic paravertebral space was stained in his cadaveric study through the porous tissue around the superior costotransverse ligament.^[17,21]^ Additionally, another study reported that there are gaps between the medial and lateral portions of the superior costotransverse ligament (fenestrations in the ligament).^[22]^ In our patient, although the contrast spreading to the spinal nerve root cannot be clearly distinguished because the echogenicity of the contrast and bone is similar, staining of the costotransverse ligament is visible. And contrast spread into the thoracic paravertebral space is visible.

In general, spread of local anesthetics in living patients is known to be more dynamic and extensive than spread in cadavers.^[23,24]^ Our contrast imaging data provides valuable information about spreading of local anesthetic and mechanism of action of ESPB from a living patient, and our report shows that ESPB can be a good option as postoperative pain control combined with multimodal analgesia after lung lobectomy. Further anatomical and clinical investigation is necessary to elucidate the detailed mechanism and clinical applications of ESPB.

## Author contributions

**Conceptualization:** Seunguk Bang.

**Data curation:** Kyudon Chung, Jihyun Chung, Subin Yoo, Sujin Baek.

**Visualization:** Sang Mook Lee.

**Writing – original draft:** Seunguk Bang, Kyudon Chung, Jihyun Chung, Subin Yoo.

**Writing – review & editing:** Seunguk Bang, Sang Mook Lee, Sujin Baek.
